# Microstrip Sensor Based on Ring Resonator Coupled with Double Square Split Ring Resonator for Solid Material Permittivity Characterization

**DOI:** 10.3390/mi14040790

**Published:** 2023-03-31

**Authors:** Khuzairi Masrakin, Siti Zuraidah Ibrahim, Hasliza A. Rahim, Saidatul Norlyana Azemi, Ping Jack Soh, Sugchai Tantiviwat

**Affiliations:** 1 Faculty of Electronic Engineering & Technology, Universiti Malaysia Perlis (UniMAP), Arau 02600, Malaysia; 2 Advanced Communication Engineering Centre of Excellence (ACE), Universiti Malaysia Perlis (UniMAP), Kangar 01000, Malaysia; 3 Centre for Wireless Communications (CWC), University of Oulu, 90014 Oulu, Finland; 4 Faculty of Industrial Education and Technology, Rajamangala University of Technology Srivijaya, Songkhla 90000, Thailand

**Keywords:** split ring resonator, permittivity, microstrip sensor

## Abstract

This paper analyzes a microwave resonator sensor based on a square split-ring resonator operating at 5.122 GHz for permittivity characterization of a material under test (MUT). A single-ring square resonator edge (S-SRR) is coupled with several double-split square ring resonators to form the structure (D-SRR). The function of the S-SRR is to generate a resonant at the center frequency, whereas D-SRRs function as sensors, with their resonant frequency being very sensitive to changes in the MUT’s permittivity. In a traditional S-SRR, a gap emerges between the ring and the feed line to improve the Q-factor, but the loss increases as a result of the mismatched coupling of the feed lines. To provide adequate matching, the microstrip feed line is directly connected to the single-ring resonator in this article. The S-SRR’s operation switches from passband to stopband by generating edge coupling with dual D-SRRs located vertically on both sides of the S-SRR. The proposed sensor was designed, fabricated, and tested to effectively identify the dielectric properties of three MUTs (Taconic-TLY5, Rogers 4003C, and FR4) by measuring the microwave sensor’s resonant frequency. When the MUT is applied to the structure, the measured findings indicate a change in resonance frequency. The primary constraint of the sensor is that it can only be modeled for materials with a permittivity ranging from 1.0 to 5.0. The proposed sensors’ acceptable performance was achieved through simulation and measurement in this paper. Although the simulated and measured resonance frequencies have shifted, mathematical models have been developed to minimize the difference and obtain greater accuracy with a sensitivity of 3.27. Hence, resonance sensors offer a mechanism for characterizing the dielectric characteristics of varied permittivity of solid materials.

## 1. Introduction

Complex permittivity is one of the fundamental electromagnetic properties of dielectric materials [1]. It is the ability of an electric field to transmit or pass through certain materials based on the characteristics of the materials themselves [2]. The dielectric property is a measure of the polarizability of a material when subjected to an electrical field. It can be represented as:

(1) $$K=\frac{\varepsilon }{\varepsilon_{0}}=\varepsilon_{r}=\varepsilon_{r}^{\prime }-j\varepsilon_{r}^{\prime \prime }$$

Permittivity can also be known as the material’s dielectric constant, which describes the interaction with an electrical field. The dielectric constant (*K*) is equivalent to the relative permittivity (*ε_r_*) or the absolute permittivity (*ε*) relative to the permittivity of free space (*ε_0_*). The real part of the permittivity (*ε_r_’*) is a measure of how much energy from an external electrical field is stored in a material, whilst the imaginary part of the permittivity (j*ε_r_’’*) is a measure of how dissipative a material can be when subjected to an electrical field, also known as the loss factor of a material [3].

Accurate dielectric measurements play an important role in applications such as manufacturing [4], processing [5], and antenna design [6]. They can be used to quantify characterizations of the electrical and magnetic properties of materials to determine changes in their density, concentration, composition, temperature, and stress–strain tensor, among others [7,8,9,10]. The available past research has considered three states of materials to quantify their permittivity, namely solid [2,11,12], liquid [13,14], and gas [15]. This research determined the capacitance value of the capacitor in a circuit when an electrical field excited the circuit and passed through the feed line to the capacitors in the resonator.

Dielectric materials can be characterized and measured by using material-dependent dielectric properties techniques such that the dielectric properties of a material govern the wave behavior in that medium [16]. Precise and accurate permittivity detection has turned into a new challenge for studies concerning microwave sensors in general [17]. Thus, many solutions have been investigated to date.

One of the options for accurate sensing of the microwave sensors is to use a metamaterial structure such as a split ring resonator (SRR) and its complementary structure, namely a complementary split ring resonator (CSRR) [18]. The customizability of metamaterial structures presents a huge advantage over the conventional sensor in that the customized structures are made to achieve higher accuracy and resolution. Furthermore, the characterization ability for both lossy and low-loss MUTs with high sensitivity, minimal effort for MUT preparation, and a non-destructive effect are among other advantages offered by the microwave sensor.

**Figure 1:**
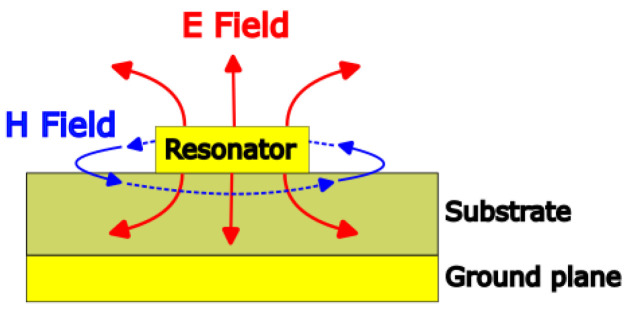
Electric (E-field) and magnetic (H-field) fields from microstrip structure.

An SRR is a common structure for obtaining negative effective permeability [19]. It shows a large magnetic dipole moment when excited by a magnetic field directed along its axis. When a magnetic field is applied along the z-axis, an electromotive force will appear around the SRR and induce currents that pass from one ring to the other through the gaps and behave as an LC circuit. Figure 1 shows the behavior of the electric field (up and down) at the microstrip design structure and a magnetic field that circulates around the conductor (copper) of the structure.

The use of the stopband reflection coefficient as a metric for substrate permittivity characterization is a relatively novel approach in the field of microstrip ring resonators. While the reflection coefficient has been used extensively to characterize the performance of microwave devices, such as filters and amplifiers, its use in the context of substrate permittivity characterization using microstrip ring resonators is a relatively new area of research. Several studies have reported the use of the transmission zero (TZ) of a transmission coefficient (S21) to characterize the permittivity of MUTs.

For example, a study in Ref. [20] proposed a novel microstrip resonator design that utilizes the stopband S21 for substrate permittivity characterization. The sensor is based on two complementary symmetric split-ring resonators (CSSRRs) and possesses a high Q-factor and wide sensing range. However, the design structure faces fabrication alignment challenges to achieve the highest accuracy, as the CSSRR is located at the bottom layer while the microstrip line is located at the top layer of the substrate.

Another study in Ref. [21] is based on a CSRR, fed by a coplanar waveguide (CPW) line on the board operating at 4.76 GHz, with the upper band sensor of a 3 × 3 CSRR array. The results showed that the resonant frequencies based on S21 TZ decreased with the increase in MUT permittivity. However, the dielectric films composed of polydimethylsiloxane and barium titanate with different volume fractions are used as the MUT (which is not common) to verify the performance of the sensor.

In a study in Ref. [22] a new microwave planar two-port circuit structure was developed as a sensor to characterize the complex permittivity of dielectric materials, based on a CSRR inserted in the circuit patch. The permittivity characterization is based on the resonant frequency of S21. Good results were obtained for sensitivity, with its best performance mainly in the range of low permittivity values. However, the experimental analysis was performed with the two MUTs only, dielectric materials FR4 and glass.

In a study in Ref. [23], they developed a Parallel Interdigital Capacitor (P-IDC)-based cost-effective microwave sensor for measuring the complex permittivity of solid materials with high accuracy and enhanced sensitivity. The results showed that the proposed sensor had a high stopband reflection coefficient for measuring the permittivity of low-loss substrates, indicating a strong resonance and high sensitivity.

In the cited literature, all these works propose a transmission coefficient, S21, to characterize the MUTs’ permittivity. Yet, the use of the stopband reflection coefficient has several advantages over other parameters, such as the *Q*-factor or the S21 parameter. For example, the stopband reflection coefficient can provide a more direct measure of the resonant frequency and the bandwidth of the ring resonator, which are important parameters for substrate permittivity characterization. Additionally, the stopband reflection coefficient can be easily measured using a one-port Vector Network Analyzer (VNA), making it a simple and convenient metric for practical applications. It was demonstrated in Ref. [24] that a one-port VNA can be replaced by a multiport reflectometer for gas concentration characterization with a planar sensor. The results of this demonstration are consistent with the theory described in Ref. [25]. The multiport reflectometer as a substitute for VNA can use narrowband sensors such as CSRR [26] or broadband sensors such as CPW [24] or coaxial probes [27], depending on the application.

Therefore, in this paper, the reflection coefficient, S11, of the stopband will be used to characterize the permittivity of a solid MUT. Overall, the use of the stopband reflection coefficient as a metric for substrate permittivity characterization using microstrip ring resonators represents a novel approach, and the mathematical model of calibration that is presented in this paper shows promise for improving the accuracy of the proposed sensors.

The performance of certain types of planar microstrip structures can be evaluated in terms of their scattering parameters, namely the reflection coefficient S11. In a conventional S-SRR, a gap appears between the ring and the feed line to achieve a better *Q*-factor, but the loss increases due to mismatched coupling feed lines. In this paper, the microstrip feed line is directly connected to the single-ring resonator to provide a good match. The SSRR’s operation switches from passband to stopband by generating edge coupling with multiple D-SRRs on both of the outer sides of the S-SRR. This paper focuses on the usage of D-SRRs to characterize permittivity using the S11 value.

## 2. Sensor Design: The Materials

### 2.1. Design of Square Resonator with Microstrip Feed Line

**Figure 2:**
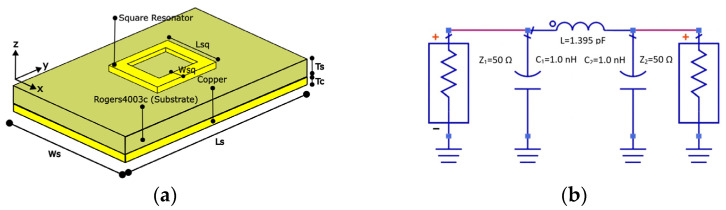
Design of (**a**) square ring resonator on a substrate and (**b**) equivalent circuit.

Precise and accurate permittivity detection has presented a new challenge for microwave sensor research in general. One of the many solutions that have been investigated to date is the use of a metamaterial structure such as a split ring resonator and its complementary structure. In this paper, a microstrip square ring resonator was designed, as in Figure 2a, based on the microstrip technique shown in Figure 1. The electric field stored in the resonator could be used to detect the MUT permittivity. The square ring was designed on a Rogers Duroid 4003C substrate(Rogers Technologies (Singapore) Inc.) with dielectric properties *(ε_r_*) of 3.38, tangent loss (*tan δ*) of 0.0027 and substrate thickness of *Ts* = 0.508 mm. The substrate’s overall dimensions are *Ws* = 35 mm width and *Ls* = 55 mm length. The square ring was centered on the substrate and occupied *Wsq* = 1.86 mm width, *Lsq* = 10.59 mm length, and a default thickness of copper cladding *Tc* = 0.017. The structure of the square ring resonator can be modeled with an equivalence LC circuit as shown in Figure 2b and its value is tabulated in Table 1.

**Figure 3:**
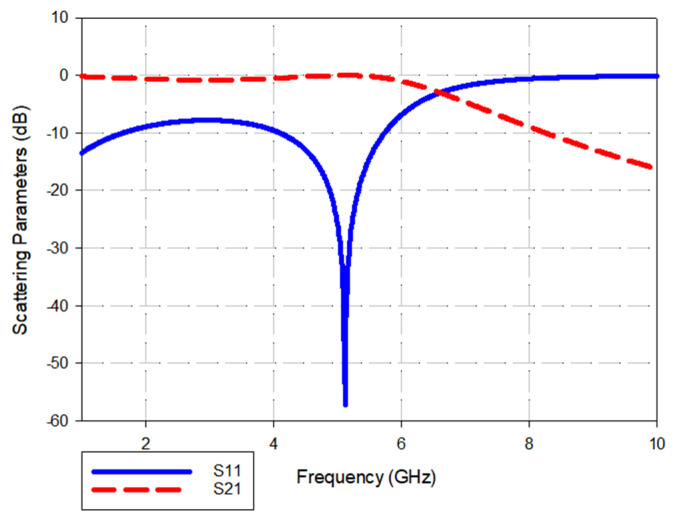
Simulation response of ADS equivalent circuit.

The equivalence circuit of Figure 2b can be obtained by using Equation (2):

(2) $$fr=\frac{1}{2\pi \sqrt{LC}}$$

where *f_r_* indicates the resonance frequency of the square resonator. The *C* denotes the total value of capacitors, and *L* denotes the value of inductor in the structure. When the circuit illustrated in Figure 2b was simulated using the circuit simulation feature of the Advanced Design System (ADS) software (2020, Keysight Technologies, Santa Rosa, CA, USA), the scattering parameters of S11 and S21 are displayed in Figure 3.

**Figure 4:**
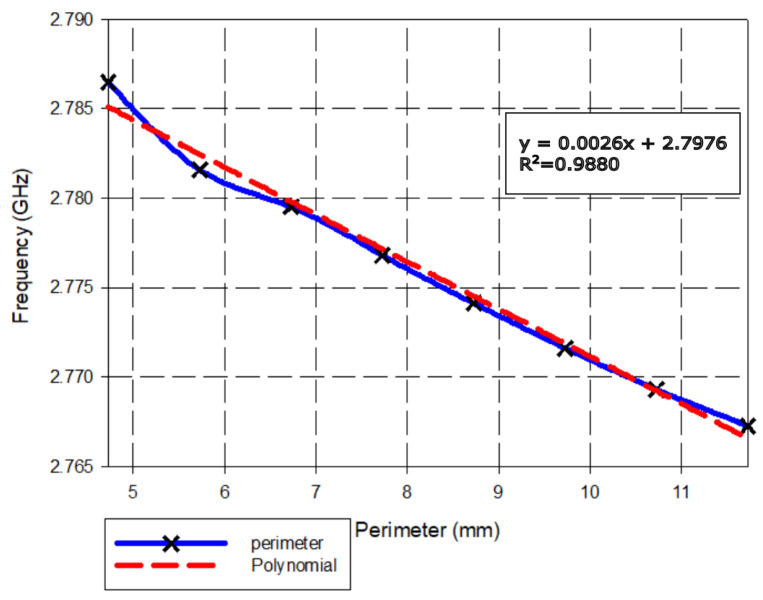
Graph of perimeter of square ring against resonance frequency and regression plot.

The customizability of such resonator structures present a huge advantage over the conventional sensor, such that the customized structures are made to achieve higher accuracy and resolution. The length of the resonator (perimeter) is among the parameters that are customized to achieve better accuracy. Figure 4 shows the correlation between the perimeter of the square ring and its resonance characteristics when simulated in the eigenmode solver in CST Microwave Studio. The simulation was created to examine the resonance property of a square ring resonator without the feed lines. The *Lsq* is varied from 1.18 mm to 3.18 mm, resulting in a total perimeter length (*Lsq* × 4) ranging from 4.72 mm to 12.72 mm, for the purpose of analyzing the trend of the resonant frequency when the resonator length was varied. The result (blue line) indicates an inverse proportion of the perimeter to the resonance frequency, such that the resonance increases when the perimeter decreases. The polynomial trend line plot (red dashed line) also suggests an inverse relationship between the two parameters.

The linear regression plot can be expressed in a mathematical as:

(3) y=−0.0026x+2.796

where R^2^ = 0.9880.

**Figure 5:**
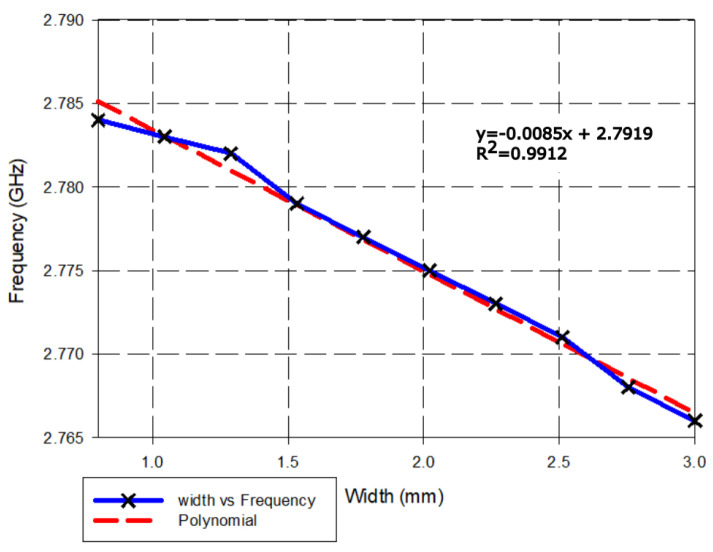
Graph of the width of square ring resonator, *Wsq* against resonance frequency and regression plot.

The sweeping eigenmode simulation was repeated while varying the width of the ring resonator, *Wsq*, and keeping *Lsq* constant at 3.18 mm. *Wsq* = 0.8 mm to 3.0 mm was considered in the parametric studies. Figure 5 shows the graph of the width of the square ring against the resonance frequency (blue line) and its polynomial trend line plot (red dashed line). The procedure was performed to seek a correlation between the width of the square ring and its resonance characteristics. The plot indicates an inversely proportional relationship between the simulated parameters, showing that the decreasing width of the square ring resonator increases the resonance frequencies.

The linear regression plot can be expressed mathematically as:

(4) y=−0.0085x+2.7919

where R^2^ = 0.9912.

**Figure 6:**
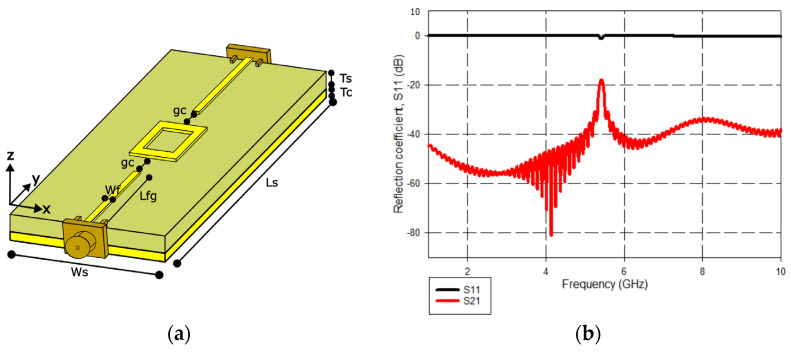
The (**a**) unconnected microstrip feed line structures and (**b**) its scattering parameter response.

Figure 6a depicts a conventional SRR with a microstrip edge-coupled feed line having a width (*Wf*) of 1.86 mm and a length (*Lfg*) of 21.81 mm. The gap between the feed lines and the resonator, *gc* was 0.4 mm and acted as a parallel plate, which is also known as the capacitance for the whole structure. The gap was made large enough to accommodate the parallel plates: if the distance was smaller than the designed gap, the capacitance value will be considered negligible. The structure was simulated in the frequency range of 1 GHz to 10 GHz, which resulted in the resonance shown in Figure 6b. The presence of a gap between the ring and the feed line results in a higher Q-factor, but the loss increases due to mismatched coupling feed lines. As a result, the performance of S21 has a high insertion loss (−17.94 dB), while the S11 shows a mismatch with an S11 value closer to 0 dB (−1.30 dB).

**Figure 7:**
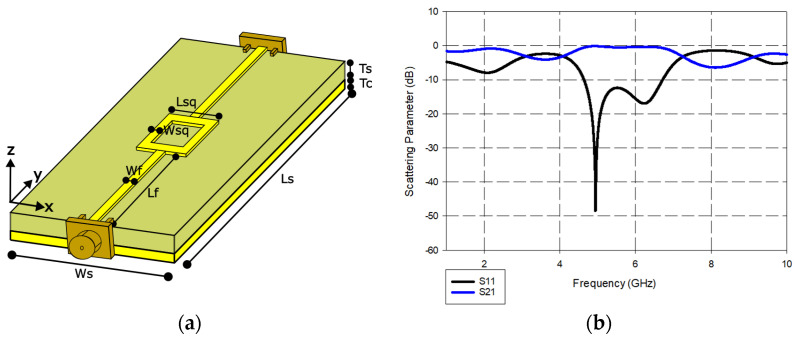
(**a**) Addition of feed lines on the substrate and (**b**) its S11 and S21 response.

Figure 7 shows the S-SRR with the microstrip transmission line connected. The width, *Wf* = 1.86 mm, is the same, but the length, *Lf* = 22.81 mm, is longer because there is no gap between the feed line and the square resonator, *gc*. Compared to the conventional structure shown in Figure 6, the microstrip feed line is directly connected to the single-ring resonator in Figure 7; consequently, providing good matching of S11. When simulated in CST Microwave Studio with two waveguide ports at the ends of the two feed lines, a resonance frequency of *fr* = 5.122 GHz with a magnitude of 48.18 dB of return loss is found, which shows that the matching works well, as shown in Figure 7b. The graph also displays the wideband characteristics of the return loss response. The magnitude of the insertion loss, S21 was at a maximum of −2 dB. Table 2 lists the optimized dimensions of the designed structure shown in Figure 7a.

**Figure 8:**
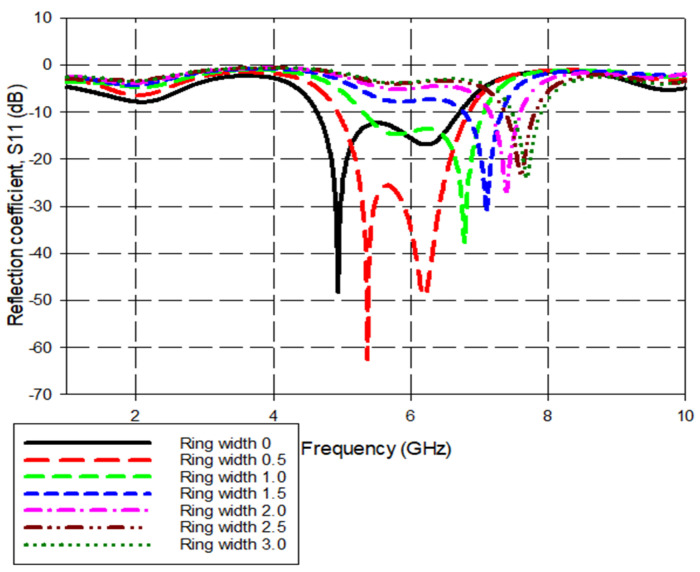
Graph of parameter sweep of ring width against resonance frequency shifting.

A parametric study was performed in an attempt to seek a correlation between the width of the ring resonator and the resonance characteristics. The ring width, *Wsq* was iteratively increased from 0.5 mm to a maximum of 3.0 mm. The results of the parametric study, as shown in Figure 8, indicate that multiple resonances occurred along the simulated frequency range of 1–10 GHz, with the maximum magnitude of insertion loss of −62.53 dB at the 5.37 GHz resonance frequency and wide bandwidth. Increasing the width showed a gradual decrease in the magnitude of insertion loss as well as the bandwidth.

### 2.2. Double Square Split Ring Resonator Sensing Principle

**Figure 9:**
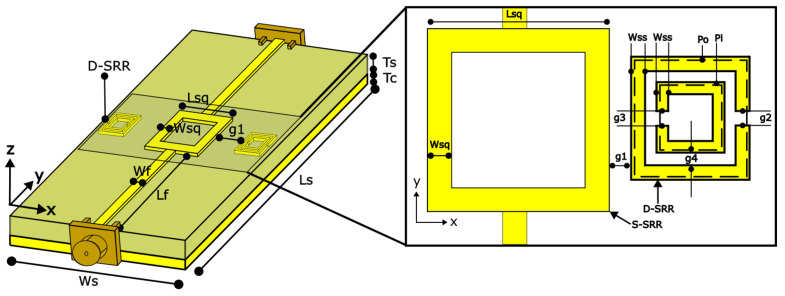
S-SRR edge coupled with D-SRR proposed structure.

Figure 9 depicts the suggested S-SRR edge-coupled D-SRR structure. Its design was inspired by [28], and it can have a square or round ring [29]. The double split square ring, or D-SRR, which was positioned on both sides of the S-SRR, served as the proposed structure’s primary sensing component. When activated by a time-varying magnetic field component in its axial direction, the D-SRR can prevent signal propagation in a region near its resonant frequency. It exhibits a quasi-static resonance. Due to its ability to resonate with a significantly smaller size and reduced radiation losses, the D-SRR provides benefits over the traditional microwave resonator.

The D-SRR was positioned in the middle of the S-SRR on both sides and is separated by *g*1 = 0.4 mm. The proposed D-SRR has two rings—an inner and an outer ring—with a copper width of *Wss* = 0.4 mm for each ring, an end gap of *g*2 = *g*3 = 0.4 mm for each ring, and a gap of *g*4 = 0.4 mm between the rings. The outer ring’s length, *Po* = 20 mm (5.0 mm × 4), while the inner ring’s length, *Pi* = 13.6 mm (3.4 mm × 4). The outer ring was positioned opposite the inner rings, causing the spaces between the two rings to lie on different planes. 

### 2.3. Simulation of Square Resonator Coupled Double Square Split Resonator

**Figure 10:**
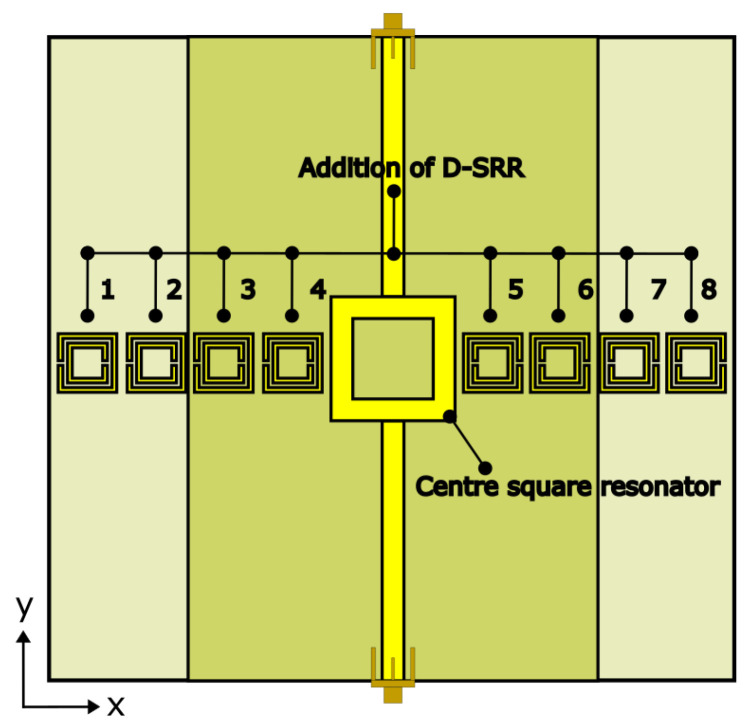
Addition of D-SRRs perpendicularly to the center resonator.

**Figure 11:**
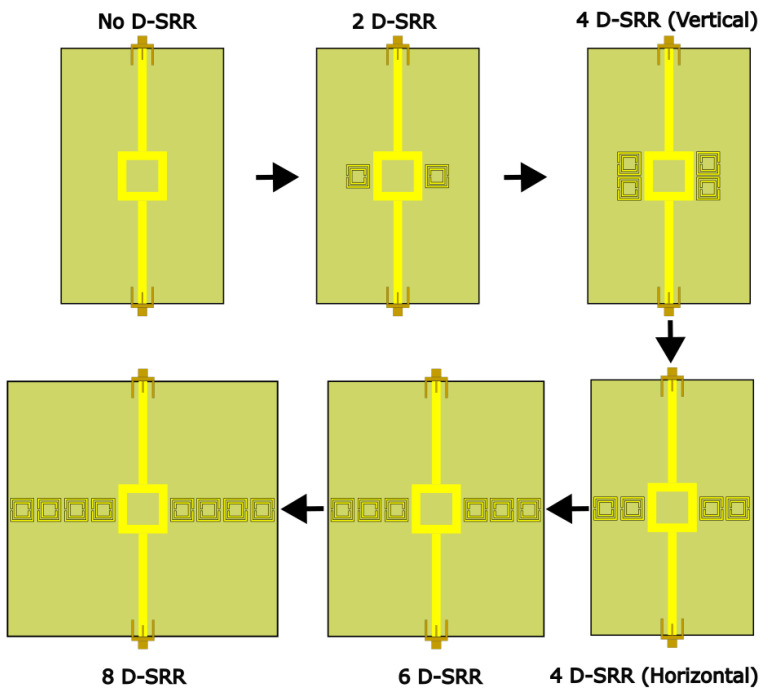
Addition of DSSRs block diagram flow process.

**Figure 12:**
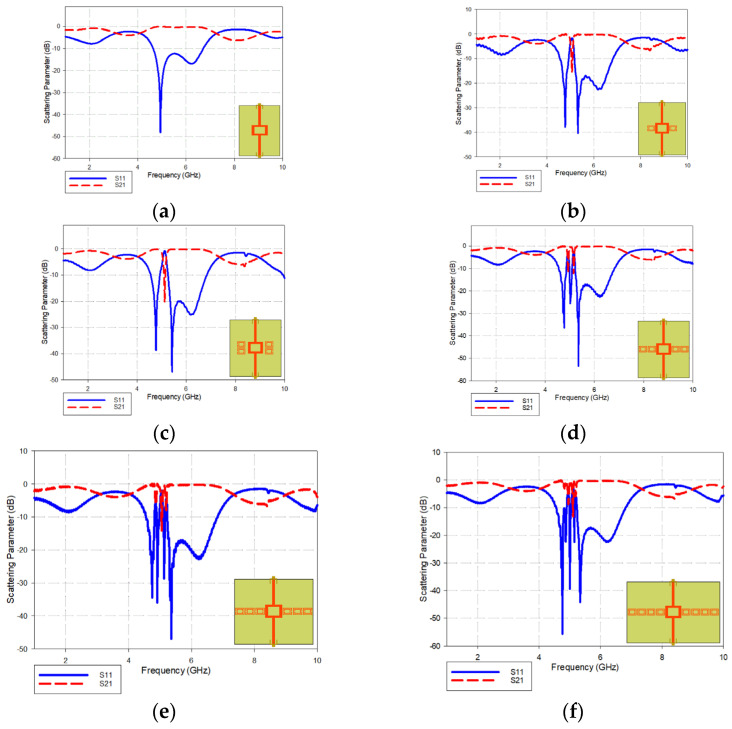
S-parameter response of (**a**) without D-SRR, (**b**) 2 D-SRRs, (**c**) 4 vertical D-SRRs, (**d**) 4 horizontal D-SRRs, (**e**) 6 horizontal D-SRRs, (**f**) 8 horizontal D-SRRs.

The designed microwave sensor was intended to offer better accuracy and resolution when analyzed in a simulation environment; therefore, a case study, such as considering the number of D-SRRs around the center resonator, was performed to seek the relationship with its resonance characteristics. The D-SRRs were placed symmetrically to the center resonator iteratively (iteration, i = 2, 4, 6, and 8), as in Figure 10, and they were separated by 0.3 mm distances. The dimension of the resonator substrate was extended to fit in a maximum of eight D-SRRs to evaluate the relationship between resonance characteristics and the number of D-SRRs, and the transition process without D-SRRs up until a maximum of eight D-SRRs is displayed in Figure 11. Figure 12 shows the correlation between the number of D-SRR reflection coefficients, S11, and the resonance frequency, where it can be clearly seen that the structure with the most promising idle performance is the one with four parallel D-SRRs. It gives a clear S11 response with less fluctuation as compared to the other simulated structures.

Figure 12 shows the simulated S-parameter results of multiple possible configurations of placing the D-SRRs. As deduced from Figure 12a, the performance of the S-SRR without the D-SRR shows passband performance with low insertion loss. Although the S11 performance shows good matching at the resonant frequency, achieving −48 dB at 5.122 GHz, it shows a wideband performance, which is not good for detecting small changes in the dielectric properties of the material under test (MUT). When D-SRRs are placed at each side of the S-SRR, remarkably, the performance switches from a passband to a stopband, as shown in Figure 12b. A narrowband S11 is achieved when reflection zeros (RZ) appear on both sides of the stopband, indicating good selectivity performances. When another D-SRR is added on each side of the S-SRR, the performance shown in Figure 12c indicates the improvement in the S11 value, where the S11 value is closer to 0 dB compared to the result shown in Figure 12b. Then, the configuration is changed from a vertical D-SRR to a horizontal D-SRR by adding D-SRRs one by one in a horizontal position, but the simulated performances shown in Figure 12d–f indicate the generation of multiple bands, which is not suitable for detecting the dielectric properties of the MUT. By analyzing this performance, it is clear that the performance shown in Figure 12c with two D-SRRs placed on both sides of the S-SRR is the best; therefore, this structure was used in the investigation with the MUT in the next section.

In CST Microwave Studio, the electric field distribution within the resonator can be visualized using the Field Calculator tool. By analyzing the electric field distribution, it is possible to identify regions with a high electric field concentration and optimize the resonator geometry to maximize the electric field in the sensing region. This can lead to improvements in the sensitivity and selectivity of the resonator-based sensor. It is important to note that, while maximizing the electric field concentration in the sensing region can enhance the sensitivity of the resonator, it may also increase the noise and reduce the overall quality factor of the resonator. Therefore, it is important to balance the design parameters carefully in order to optimize the sensor performance for a specific application.

**Figure 13:**
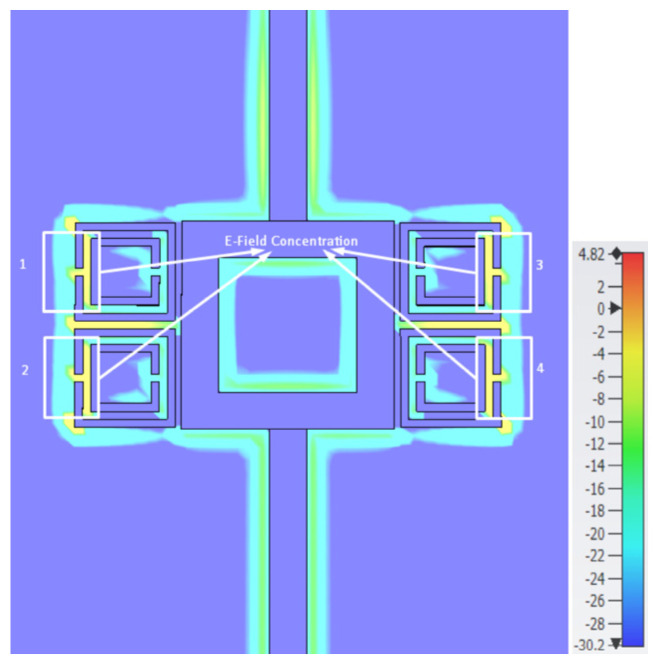
Simulation of E-field concentration at *fr* = 5.122 GHz (The highlighted region 1-4 shows a yellow color, indicating regions of high electric field concentration that are sensitive to the small changes in MUT).

To maximize the electric field concentration in the sensing region of a D-SRR, several design strategies can be employed. One approach is to adjust the resonator geometry to optimize the coupling between the resonant mode and the sensing material. For example, the gap between the S-SRR and the D-SRR can be adjusted to maximize the electric field overlap between the two regions. Therefore, it is important to investigate the E-field distribution of the proposed structure. Figure 13 shows the electric field distribution using contour maps at the surface of the proposed sensor, performed via CST at the resonant frequency at its maximum value of the electrical field. The E-field distribution is performed on the structure with four D-SRRs at the resonant frequency, 5.122 GHz. The boundary is set as open in the top direction with 5*substrate thickness and in the bottom direction with 1*substrate thickness of the structure. The signal excitation is performed by using a waveguide port. As seen in Figure 13, the D-SRR shows a yellow color, indicating regions of high electric field concentration that are sensitive to the small changes in MUT.

Figure 13 shows the electric field distribution at the surface of the proposed sensor, performed via CST at the resonant frequency. The MUT sample can be placed at the center resonator while covering all of the D-SRR, where, during the resonance, an electric field is established across the gap between the D-SRR capacitive plate and the square resonator, making the region near and inside the D-SRR sensitive to dielectric changes. Therefore, it is possible to use this region inside the D-SRR to measure the dielectric properties of materials.

**Figure 14:**
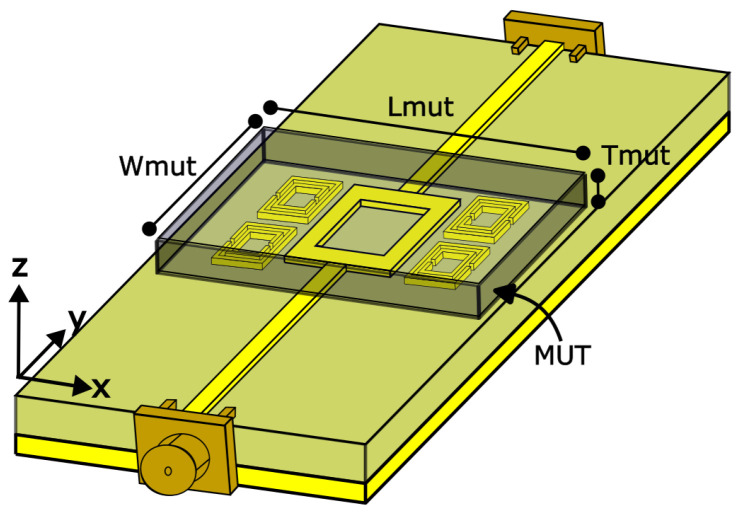
Proposed sensor with MUT on the top.

After the aforementioned case study, a parametric study was conducted using CST Microwave Studio simulation to examine the relationship between resonance characteristics and a range of permittivity, *ε_r_*, of MUT. As shown in Figure 14, the MUT with the dimensions *Wmut* × *Lmut* and *Tmut* is put on top of the resonator, covering the entire region of the S-SRR and four parallel D-SRRs. The research made the assumption that the permittivity value could be determined through scattering parameter response shifting when the MUT was mounted on top of the resonator, based on the E-field concentration behavior. 

## 3. The Resonant Frequency Analysis

**Figure 15:**
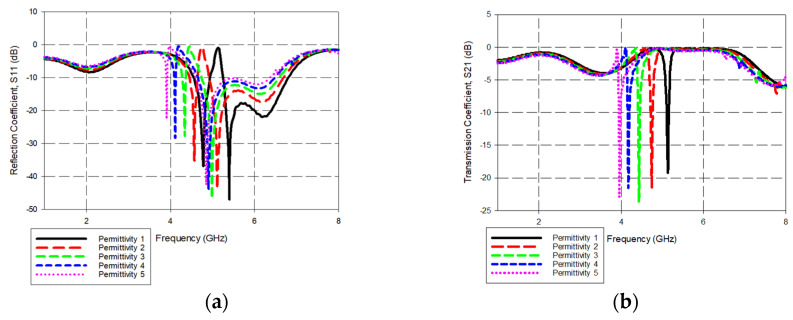
Shifting in resonance of (**a**) reflection coefficient (S11) and (**b**) transmission coefficient when simulated with a range of 1−5 dielectric constant.

The designed resonator structure underwent several frequency-permittivity analyses in order to verify its performance with the potential physical MUTs that were to be introduced to the structure. A set of dielectric constants ranging from 1–5 was simulated to evaluate shifts in the resonance frequency, *fr* as depicted in Figure 15. The simulation trend showed a shift to the left when a larger permittivity was introduced. The peak resonance frequency did not seem to change, but the overall response decreased when a larger permittivity was introduced.

**Figure 16:**
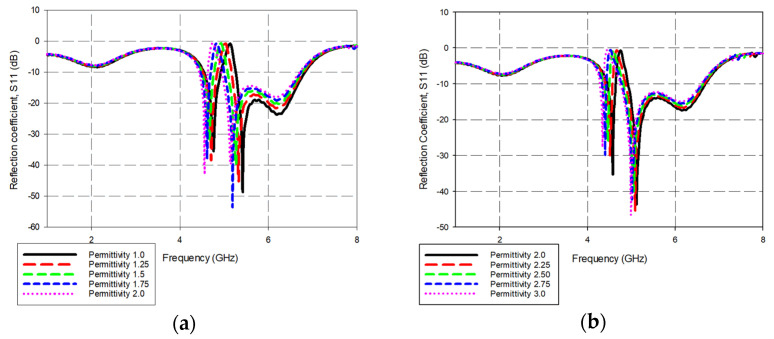

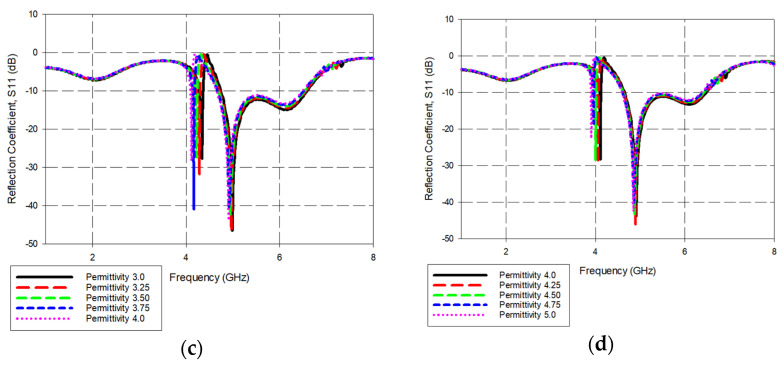
The graph of small range of permittivity simulation (**a**) 1–2 (**b**) 2–3 (**c**) 3–4 (**d**) 4–5.

The procedure of simulating ranges of permittivity was replicated and analyzed with smaller values with the same objective. Ranges were divided into four different sets (1–2, 2–3, 3–4, 4–5), which are shown in Figure 16a–d.

**Figure 17:**
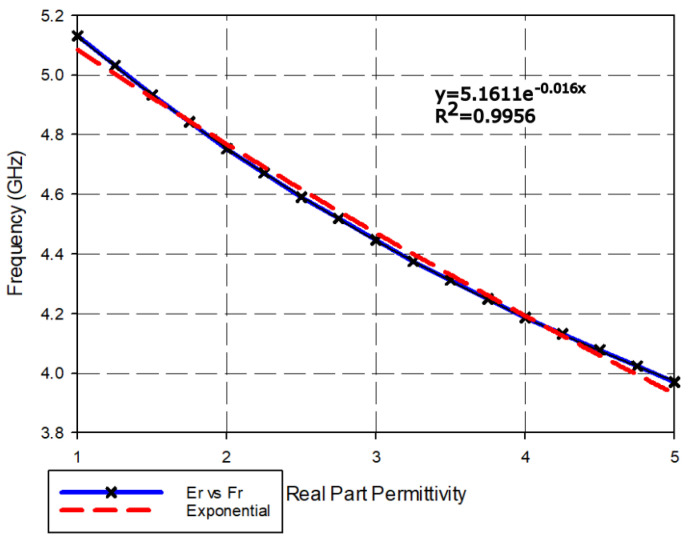
Plotted graph of permittivity versus shifted frequency when S11 is at its peak value.

As can be seen in Figure 16, when the dielectric constant changes from 1 to 5 with a scale of 0.25, the frequency shifts to a lower frequency range with reference to the peak value of S11. The relation between the dielectric constant and the frequency shift is plotted, as shown in Figure 17.

The permittivity of a microstrip ring resonator can be modeled as an exponential function of the resonant frequency. The exponential model for the permittivity of the microstrip ring resonator is given by:

(5) $$y=5.1611e^{-0.016x}$$

where *y* is the resonant frequency and *x* is the permittivity.

The exponential model is based on the fact that the permittivity of the microstrip ring resonator changes with frequency due to the presence of losses in the material. The losses cause the permittivity to decrease with increasing frequency, which is captured by the exponential term in the equation.

The relaxation time *x* in the exponential model is a measure of the time it takes for the material to respond to changes in the electric field. It is related to the loss of tangency of the material and the conductivity of the substrate. The value of *x* can be determined experimentally by measuring the permittivity at different frequencies and fitting the data to the exponential model.

Overall, the exponential model provides a useful way to describe the permittivity of a microstrip ring resonator as a function of frequency and can be used to design and analyze microstrip ring resonators for various applications in microwave engineering.

Next, the quality factor of the proposed structure is evaluated using Equations (6) and (7).

(6) $$\Delta f=\frac{f_{1}}{f_{2}}$$

(7) $$Q=\frac{f_{r}}{\Delta f}=\frac{1}{\tan\delta }$$

**Figure 18:**
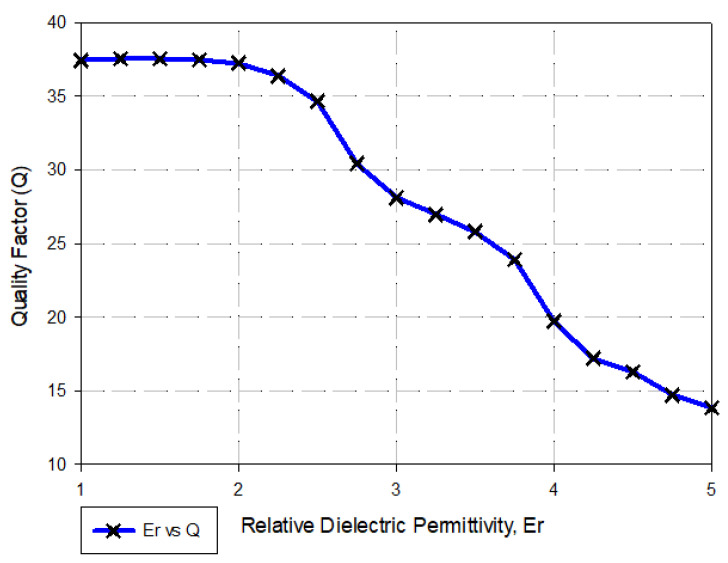
Plotted graph of relative permittivity vs. *Q*-factor.

*Δf* in Equation (6) represents the frequency range between *f_1_* and *f_2_*. *f_1_* and *f_2_* are obtained by measuring −3 dB from the peak value of S11. The symbol *f_r_* in Equation (7) represents the frequency value when S11 is at its peak value, while *tan δ* represents the tangent loss. The calculated values of Equations (6) and (7) are shown in Table 3. Figure 18 shows the plotted graph of permittivity versus the *Q*-factor shown in Table 3.

As seen in Figure 18, the highest Q-factor is observed for low permittivity values between 1 and 2. The Q-factor started decreasing when permittivity increased. The relationship shows that the proposed sensor works best for characterizing MUTs with a permittivity between 1 and 2.

**Figure 19:**
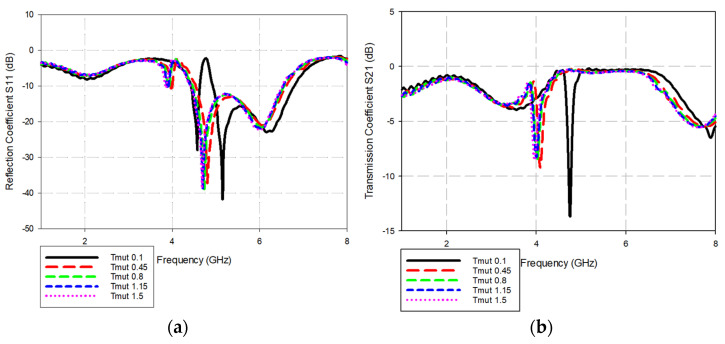

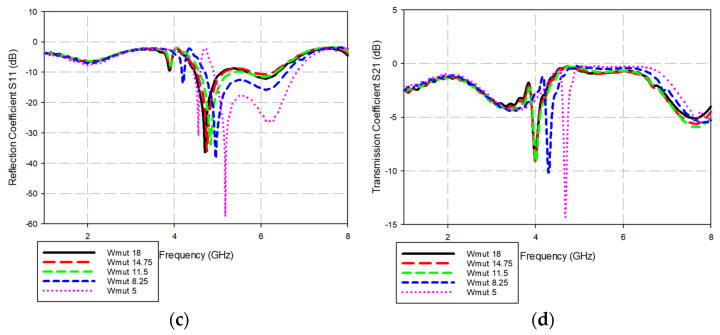
(**a**) Reflection coefficient S11 of different thicknesses of MUT, (**b**) transmission coefficient S21 of different thicknesses of MUT. (**c**) Reflection coefficient S11 of different widths of MUT, (**d**) transmission coefficient S21 of different widths of MUT.

Figure 19 shows the simulated S-parameters of the parametric study when the MUT of the FR4 substrate varies by thickness (*T_MUT_*) and width (*W_MUT_)*. As observed in Figure 19a,b, significant changes in S11 and S21 can be noticed when *Tmut* = 0.1, while the response of S11 and S21 is similar for other thicknesses. This observation can indicate that the near-field interaction with the MUT may include the air in the region above the MUT, hence, to achieve better accuracy, the MUT thickness should be greater than 0.1 mm. As shown in Figure 19c,d, the significant changes in S11 and S21 can be noticed when *Wmut* = 5 and *Wmut* = 8.25. It is noted with these *Wmut* values that the MUT size is smaller and does not cover the whole area of the D-SRR. Therefore, the S11 and S21 shift significantly as the behavior of the near-field electric propagation is different when the MUT size is smaller than the D-SRR size.

**Figure 20:**
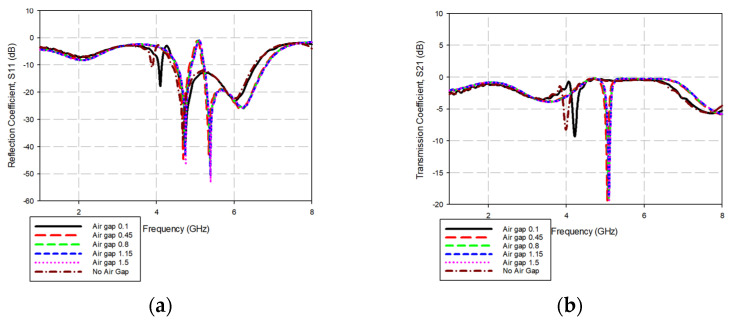
The air gap simulation response of (**a**) reflection coefficient and (**b**) transmission coefficient between MUT and resonator.

Figure 20 shows the simulated performance when the air gap is considered between MUT (FR4 with a *Tmut* = 1.6 mm) and the proposed sensor. As observed in Figure 20, significant changes in S11 and S21 can be noticed when the air gap is at 0.45 and above, which resembles the performance of free space with the resonant frequency shifted to the higher frequency. Therefore, the presence of an air gap between the sensor and MUT will affect the resonant frequencies of S11 and S21.

## 4. Result and Discussion

In order to verify the performance of the proposed microwave sensor for the characterization of MUT samples and to illustrate the relationship between the resonance frequency and the complex permittivity of the MUT samples, the microwave sensor was fabricated using standard chemical etching techniques.

The fabrication procedures were initiated with film processing, where two layers of film were laminated on the ground plane and top resonator plane for a plot tracing photo from the Gerber film to the Rogers 4003C board. The laminated board was exposed to ultraviolet (UV) rays for 30 s in a vacuum exposure unit to harden the resists before removing the outer film layers (negative and protective layers). The UV exposure needed to be performed in a dark room to eliminate unnecessary light disturbances. Removing negative and protective layers after the UV process revealed photoresists tracing on the substrate board. It was then transferred to the inlet of the developer machine to remove the unexposed photoresist part of the board with potassium carbonate solution. The developing process could take several minutes and was repeated to ensure that the unwanted tracing has been completely removed. Next, it was inserted into the photo stripper machine for 3 min to remove unwanted photoresist films. Once the process had finished, the board was spray washed with tap water before being dried by a hot plate. Once the board was dried, it was cut following the dimensions that had been designed previously in the software. The board was ready for measurement when the SMA (SubMiniature A) connector was mounted at the edge of the board that connected to the microstrip transmission line.

**Figure 21:**
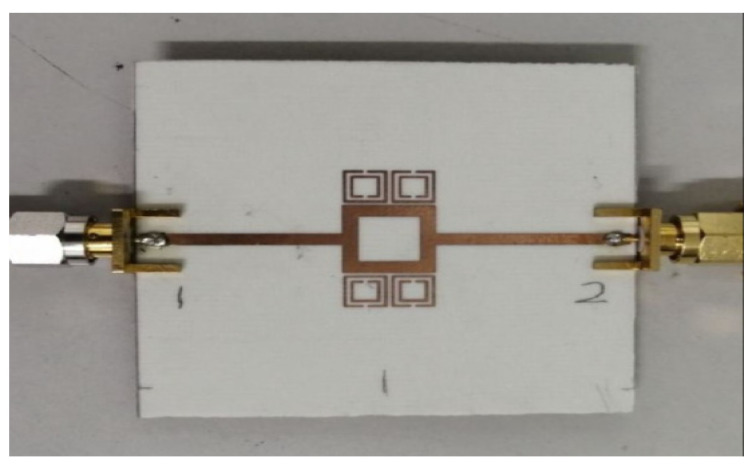
Prototype of the proposed sensor.

The fabricated structure of the microwave sensor is shown in Figure 21. Rogers Duroid 4003C with a 0.508 mm thickness, 0.0027 loss tangent, and 3.38 dielectric constant was chosen as the substrate board in the fabrication process. Rogers Duroid 4003C also comes with 17 μm of electrodeposited copper foil thickness to construct the structure of the microwave sensor while the bottom side of the board features a full ground plane of copper. A pair of 50 Ω impedance SubMiniature version A (SMA) connectors were soldered and mounted on the feed line at both ends of the fabricated microstrip resonators, as in Figure 21.

**Figure 22:**
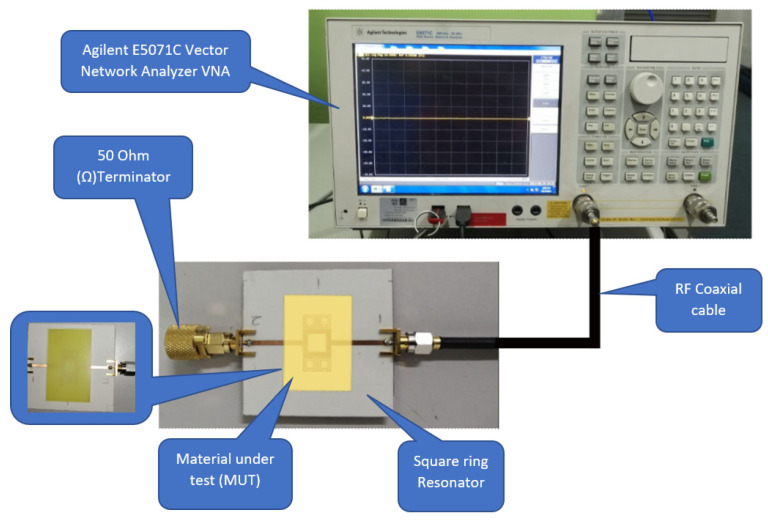
Measurement setup of MUT samples.

The resonator was then fed with an electromagnetic signal through an electromagnetic coaxial cable connected with an E5071C Agilent VNA port 1 (Agilent Technologies, Santa Clara, CA, USA). Three pieces of known dielectric permittivity, namely Taconic TLY-5 (Korea Taconic, Korea), FR4 (Jiujiang PTFE Materials Co., Ltd. Jiujiang, China), and Rogers Duroid 4003C, were chosen as the samples to be measured. The samples were simply placed on the center top of the resonator covering all of the ring structures in order to accurately quantify the permittivity. The dimensions of the MUTs used in the measurements and in simulations are the same for precise quantification and are tabulated in Table 4. The measurement was considered its reflection (S11) parameters, such that the measurement setup was performed using VNA with one coaxial cable connected to port 1 of the designed resonator, while the port at the other end was terminated with 50 Ω impedance, as displayed in Figure 22.

**Figure 23:**
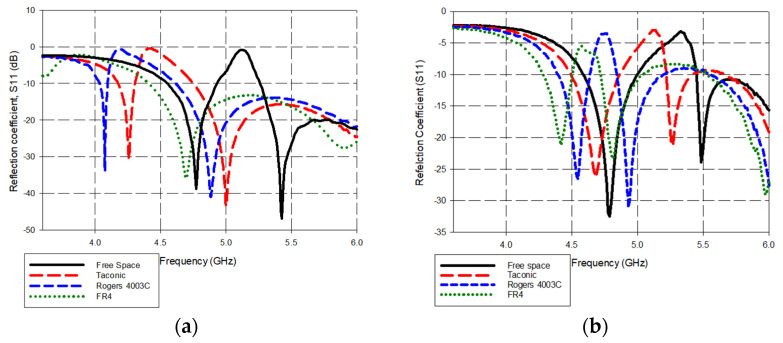
Reflection coefficient of the sensing system sensor for different MUTs: (**a**) Simulated CST; (**b**) measured.

Figure 23 shows the results for the proposed sensors, both simulated (CST) and measured (VNA).

There is a good agreement between the trends of both cases, where the resonant frequency shifts to the lower frequency when permittivity decreases, although some frequency shift is observed between the simulated and the corresponding measured results. There could be several reasons why there is a discrepancy between the simulated and fabricated performances of the proposed sensor. Firstly, a possible reason is dimensional accuracy. The resonant elements in the D-SRR must be fabricated with high precision to ensure that they resonate at the desired frequency. Deviations from the desired dimensions can lead to shifts in the resonant frequency or changes in the bandwidth of the resonant response. Secondly, the substrate thickness and dielectric constant could be possible causes. The thickness and dielectric constant of the substrate can impact the properties of the D-SRR, such as the resonant frequency and the bandwidth of the resonant response. Deviations from the desired substrate thickness or dielectric constant can result in changes to the D-SRR properties. Thirdly, alignment accuracy can also cause the discrepancy between the simulated and measured results of the fabricated D-SRR. The resonant elements in the D-SRR must be aligned with high precision to ensure that they resonate coherently and produce the desired D-SRR response. Misalignment between the resonant elements can lead to reduced coupling and weaker resonant responses. Finally, fabrication errors may be the reason. Errors in the fabrication process, such as non-uniform etching or deposition, can impact the properties of the D-SRR and introduce additional scattering or loss.

Simulations and measurements can sometimes produce different results for substrate samples, and the differences between the two can vary in magnitude. Simulations are limited by the accuracy and complexity of the models used to represent the substrate sample. For example, a simulation may not include all relevant physical phenomena or may not capture the geometry of the substrate sample accurately. It is assumed that there is no air gap when placing the MUT on top of the sensor in the simulation. In addition, it is assumed that the substrate has a flat surface and a constant thickness. However, when placing the MUT in real environment, it is difficult to make sure that there is no air gap between the MUT and the sensor. These modeling limitations can contribute to differences between the simulated and measured results. The measured substrate samples may have natural variations in their material properties that are difficult to capture in simulations. This can lead to differences in the measured results compared to the simulated results, which may assume idealized material properties. Measurement results can be affected by experimental errors, such as measurement noise, calibration errors, or variations in environmental conditions. These factors can contribute to measurement uncertainty, which can affect the accuracy and precision of the results.

**Figure 24:**
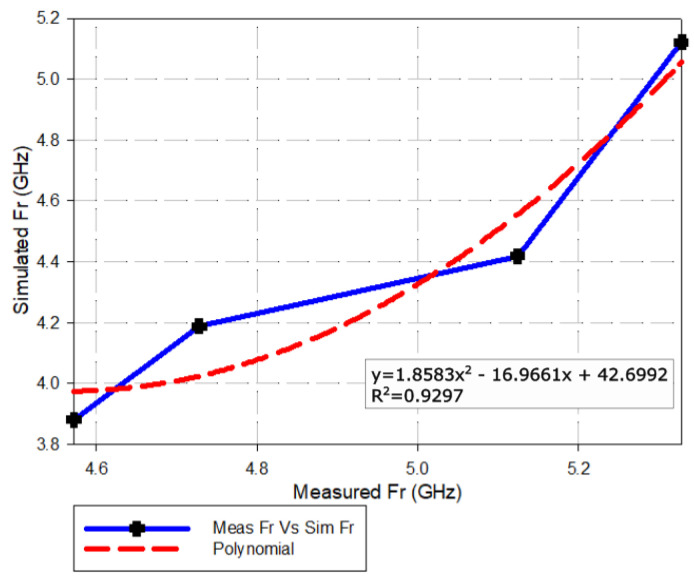
Difference of simulated against measured resonance frequency of the MUT.

Since it is shown that there are different values for the simulation and measurement results, for this reason, the resonant frequency from measurement (*f_m_*) and simulation results (*f_s_*) must be calibrated and used as a reference in determining the value of the permittivity. Figure 24 shows the fitting curve of both the resonant frequency from the simulation process and the measured results that can be expressed by a mathematical model as follows:

(8) $$fr_{cal}=1.8583f_{m}{}^{2}-16.9661f_{m}+42.6992$$

Furthermore, the calibrated frequency from the proposed resonator is obtained using Equation (8), as shown in Table 5, where *fr_cal_* is the calibrated resonant frequency. *fr_cal_* can be used to determine the permittivity value of the MUT.

**Figure 25:**
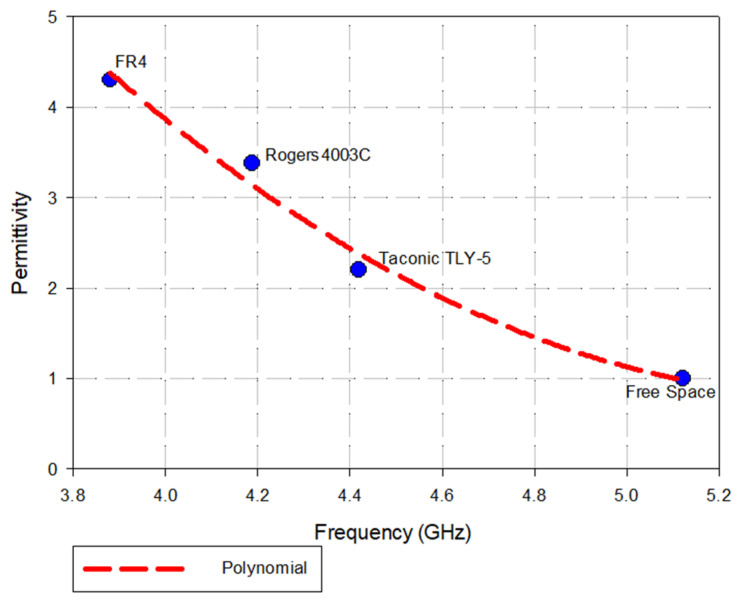
Calibrated frequency (GHz) against permittivity.

To determine the permittivity of the MUT, the resonant frequency from the simulation results and the reference permittivity were used to obtain a mathematical model of the fitted curve with a second-order polynomial, as shown in Figure 25.

It should be noted that the reference permittivity used in the simulation process has the same value as the permittivity from the datasheet. Figure 25 shows the fitted curve of the resonant frequency from the simulation process and reference permittivity that can be expressed by a mathematical model as follows:

(9) $$\varepsilon_{r}=1.3853fr_{cal}{}^{2}-15.2081fr_{cal}+42.5337$$

Furthermore, the permittivity value of the MUT can be determined using the calibrated resonant frequencies *fr_cal_* from Equation (8) in Equation (9).

Table 6 displayed the state-of-the-art table of comparison between past research and this work with unloaded measured quality factors using Equations (6) and (7). The sensitivity was calculated using the following mathematical Equation (10):

(10) $$S=\frac{\left|f_{\varepsilon r}-f_{unloaded}\right|}{f_{unloaded}}\times \frac{1}{\varepsilon_{r}-1}\times 100\;\%$$

In this paper, simulations and measurements were performed, achieving the acceptable performance of the proposed sensors. Although the simulated resonant frequency and the measured resonant frequency shifted, the mathematical models were established to minimize the differences and to achieve better accuracy. As deduced in Table 6, the performance of the proposed sensor is comparable to the work presented in Refs. [20,21,22,27,28]. To the best of the authors’ knowledge, this is the first solid material permittivity characterization that depends on the reflection coefficient for stopband performance. The benefit of using the S11 parameter is that it can be measured using a one-port VNA or can be substituted by a customized design of a six-port structure. The accuracy can be enhanced further using a calibration algorithm developed for a six-port structure.

## 5. Conclusions

The single-ring square resonator (S-SRR) edge-coupled with several D-SRRs was successfully presented in this study. The investigation began with a single square ring resonator with no feed line to determine its resonant frequency. When the microstrip feed line was connected directly to the single ring resonator, the matching performance and insertion loss improved over the conventional S-SRR. Surprisingly, edge coupling with multiple D-SRRs placed vertically on both outer sides of the S-SRR changes its operation from passband to stopband. The S11 parameter was used instead of the well-known S21, which provides the benefits of one-port measurement. The resonance frequency sensitivity was also improved, with the shift visible even with minor changes in permittivity. The proposed sensor was designed, built, and tested to accurately identify the dielectric properties of three MUTs (Taconic-TLY5, Rogers 4003C, and FR4). When the MUT was applied to the structure, the measured results showed a change in the resonance frequency. To deal with the difference between simulated and measured resonant frequencies, a calibration expression is presented. Although the sensor’s main limitation is that it can only be modeled for MUTs with a permittivity ranging from 1 to 5, the best performance can be observed between 1 and 2, where the Q-factor is the highest.

## Figures and Tables

**Table 1:** ADS simulation parameter for LC equivalent circuit.

Parameter	Value
Input impedance, *Z*_1_	50.0 Ω
Output impedance, *Z*_2_	50.0 Ω
Capacitor, *C*_1_	1.0 nH
Capacitor, *C*_2_	1.0 nH
Inductor, *L*	1.395 pF
Resonance frequency, *f_r_*	5.122 GHz

**Table 2:** Dimensions of the designed microstrip square ring resonator shown in Figure 7a.

Parameters	Value (mm)
Width of substrate (*Ws)*	35.0
Length of substrate *(Ls)*	55.0
Thickness of substrate *(Ts)*	0.508
Thickness of copper *(Tc)*	0.017
Width of feed line *(Wf)*	1.860
Length of feed line *(Lf)*	22.21
Length of square resonator *(Lsq)*	10.587
Width of square resonator *(Wsq)*	1.860

**Table 3:** Quality factor for the designed structure.

Permittivity (*ε_r_*)	Resonance Frequency (*f_r_*)	*f_1_*	*f_2_*	Δ*f*	Quality Factor (*Q*)
1.00	5.1310	5.0560	5.1929	0.1370	37.4526
1.25	5.0320	4.9545	5.0885	0.1340	37.5522
1.50	4.9330	4.8597	4.9911	0.1314	37.5419
1.750	4.8430	4.7708	4.9000	0.1292	37.4845
2.00	4.7530	4.6873	4.8149	0.1276	37.2492
2.25	4.6720	4.6078	4.7362	0.1284	36.3863
2.50	4.5910	4.5333	4.6657	0.1324	34.6752
2.75	4.5190	4.4618	4.6103	0.1485	30.4310
3.00	4.4470	4.3930	4.5512	0.1581	28.1278
3.25	4.3750	4.3282	4.4904	0.1622	26.9729
3.50	4.3120	4.2651	4.4321	0.1670	25.8204
3.75	4.24899	4.2048	4.3825	0.1777	23.9111
4.00	4.1860	4.1471	4.3593	0.2122	19.7267
4.25	4.13199	4.0912	4.3312	0.2400	17.2167
4.50	4.07800	4.0376	4.2880	0.2504	16.2859
4.75	4.02400	3.9867	4.2602	0.2735	14.7130
5.00	3.97000	3.9363	4.2233	0.2870	13.8327

**Table 4:** MUT samples and their dimensions.

Board	Thickness (mm)	Dielectric Constant, ε	Loss Tangent, δ	Dimension (mm)
Free space (air)	-	1.0	0	-
Taconic TLY-5	1.57	2.2	0.0009	32 × 25
FR4	1.60	4.3	0.017	32 × 25
Rogers Duroid 4003C	0.508	3.38	0.0027	25 × 25

**Table 5:** Measured frequency, simulated frequency, and calibrated frequency of the proposed square ring resonator.

Reference Permittivity (*ε_r_*)	*f_s_* (GHz)	*f_m_* (GHz)	*fr_cal_* (GHz)
1.00	5.1220	5.3280	6.189
2.20	4.4184	5.1240	5.998
3.38	4.1880	4.7280	5.651
4.30	3.8808	4.5720	5.523

**Table 6:** State-of-the-art table.

Ref.	Model	Permittivity Range, *ε_r_*	(MUT)	Resonance frequency, *f_r_* (GHz)	*Q*-Factor (3 dB)	Sensitivity, *S* (%)
[22]	CSRR (Circular)	4.25–5.37	Solid	2.65	53.97	2.86
[30]	CSRR (Square)	1.0–2.0	Solid	5.41	142	6.50
[20]	CSRR (Circular)	1.0–1.19	Solid	5.35 and 7.99	38.21	3.32
[21]	CSRR (Circular)	2.8–2.68 and 5.00	Solid	4.76	19.04	9
[31]	CSRR (Circular)	1.83–2.49	Solid	2.24	1119	0.14
This work	SRR (Square)	1.0–4.3	Solid	5.122	37.41	3.27

## Data Availability

Not applicable.
